# Supplementary material for: Unsupervised multi-scale clustering of single-cell transcriptomes to identify hierarchical structures of cell subtypes
Source: Res Sq. 2024 Dec 23:rs.3.rs-5671748. Preprint. [Version 1] doi: 10.21203/rs.3.rs-5671748/v1 (PMC11703337; doi:10.21203/rs.3.rs-5671748/v1)
Supplement: Supplement 1 [file NIHPPRS5671748v1-supplement-1.pdf]

Supplementary Files

This is a list of supplementary files associated with this preprint. Click to download.

- SUPPLEMENTARYMATERIAL.v2.docx
- SupplementaryData1.xlsx
- SupplementaryData2.xlsx
- SupplementaryData3.xlsx
